# Barriers to higher education: commonalities and contrasts in the experiences of Hindu and Muslim young women in urban Bengaluru

**DOI:** 10.1080/03057925.2016.1220825

**Published:** 2016-09-20

**Authors:** Biswamitra Sahu, Patricia Jeffery, N. Nakkeeran

**Affiliations:** ^a^ Social Sciences, Indian Institute of Public Health – Bangalore Campus, Bangalore, India; ^b^ Sociology, School of Social and Political Science, University of Edinburgh, Edinburgh, UK; ^c^ Social Sciences, Indian Institute of Public Health – Gandhinagar Campus, Sardar Patel Institute Campus, Ahmedabad, India

**Keywords:** Muslim, Hindu, women’s higher education, urban India

## Abstract

Gender inequalities in educational attainment have attracted considerable attention and this article aims to contribute to our understanding of young women’s access to higher education. The article is based on our in-depth interviews with 26 Hindu and Muslim young women attending colleges in urban Bengaluru (formerly Bangalore), south India, and explores the barriers they confronted in fulfilling their aspirations. We highlight the similarities amongst the young women, as well as the distinctive experiences of the Hindu and Muslim interviewees. Financial constraints, lack of safety for women in public space, and gender bias, gossip and social control within the family and the local community affected Hindu and Muslim interviewees in substantially similar ways. For the Muslim interviewees, however, gender disadvantage was compounded by their minority status. This both underlines the importance of incorporating communal politics into our analysis and undermines popular discourses that stereotype Muslims in India as averse to girls’ and young women’s education.

## Introduction

Indian policy documents often valorise modern education as a magic wand that will ensure ideational change among the educated and automatically create the egalitarian society that is constitutionally endorsed (Page 2005). Such high expectations placed on modern education, however, seem unrealistic in the Indian context, where access to education is very unevenly distributed across caste, class, religion and gender lines (Jeffery 2005). There is extensive discussion of various structural barriers that affect girls’ access to education (Jeffery and Jeffery 2002) and that are reflected in gendered inequalities in educational outcomes. Understanding these barriers is crucial: unless we know what curbs girls’ chances of being schooled in their formative years, we cannot explain their life-chances in later life (Drèze and Sen 2013, 58–65).

Muslims are India’s largest religious minority, yet data such as those from the census and the National Sample Survey Organisation (NSSO) indicate that Muslims occupy India’s economic, social and political margins. Muslims are poorer (NSSO 66th Round 2009–2010) and less often employed in the formal sector, which is generally better remunerated than informal-sector employment (Hameed 2005, 197–215; Mohammad-Arif 2012; NSSO 66th Round 2009–2010; Sachar 2006). For Muslim girls, gender-based barriers to higher education are compounded by their minority status and Muslim women’s access to higher education and their educational attainments are generally lower than those of Hindu caste women.

In rural India, the disadvantage of women from marginalised Hindu groups (Scheduled Castes [SCs] and Scheduled Tribes [STs]) in higher education is comparable with that of Muslim women (NSSO 66th Round 2009–2010). The SCs (also known as Dalits)^1^ occupy disadvantaged positions in regard to literacy, economic condition, domestic electricity connections and so forth compared with Other Backward Castes (OBCs) and the caste Hindus (Srinivasan and Kumar 1999). Even after controlling for other variables, statistical analysis shows that SCs and OBCs are less likely to attend school than caste Hindus (Drèze and Kingdon 2001). SC girls experience double discrimination due to their positioning as SC and as women (Paik 2009). Paik further argues that the social structure not only impedes an SC girl’s access to education but also the quality of education she receives. In this article, however, we do not address this highly significant aspect of educational marginalisation because our primary focus is on Muslim women and their problems in accessing quality higher education, which is mainly located in urban India.

In urban India, almost 72% of Muslim young women are literate, but only 7.6% of urban Muslim women have had graduate-level education (NSSO 66th Round 2009–2010). The disaggregated figures for urban Hindu women educated to graduate level and above are caste Hindus 31.3%, Hindu OBCs 16.3%, Hindu SCs 11.5% and Hindu STs 13.4%, indicating that Muslim young women are even more disadvantaged in higher education than the deprived social categories among their Hindu counterparts (Hindu SC/ST) (NSSO 66th Round 2009–2010). Multivariate analysis further highlights that Brahmin and high-caste Hindus enjoy greater educational achievements than Hindu SCs, whilst the most disadvantaged are Muslims and STs (Borooah 2012).

This greater disadvantage of urban Muslim women in higher education is contrary to expectations, because there is generally a positive association between urban location and women’s education. Hasan and Menon (2004, 18–46) stress the need for research on the low representation of Indian Muslim young women in higher education. We shall discuss in more detail later the probable reasons behind this anomaly: informal-sector employment, infrastructural disadvantages due to poverty, and Muslim boys’ low levels of educational attainment.^2^

After profiling higher education in India and outlining our research methods, we analyse our qualitative interviews with Hindu and Muslim young women pursuing college education in urban Bengaluru. We outline the value that Hindu and Muslim participants alike placed on their attainment of modern education. Then we focus on the barriers to continuing in post-school education that confronted these young women, particularly financial constraints, lack of safety for women in public space, and gender bias, gossip and social control within the family and the local community. We highlight the similarities amongst young women, irrespective of caste and religious community background, as well as the distinctive experiences of the Hindu and Muslim interviewees that underline the importance of incorporating communal politics into our analysis. Finally, we conclude that socio-economic standing, access to which is influenced by communal position, determines the educational experiences of these women.

## Higher education in India

Some crucial aspects of the Indian higher education system provide a backdrop to our discussion. Until the 1980s, higher education in India was primarily (although not exclusively) a public-sector enterprise that charged low or even no fees.

Writing about government schools, Dyson et al. (2009) and Drèze and Sen (2013) contend that they are ill-equipped to offer quality education because of teacher shortages, teacher absenteeism and teachers’ short-tenure contracts. For instance, Drèze and Sen (2013, 119) have estimated that the shortcomings plaguing government schools reduce actual teaching time to a quarter of what it should be. Teachers often prefer not to teach in government institutions because salaries there are lower than in private educational institutions. The quality of higher education has not received enough attention (Umashankar and Dutta 2007), although Dukkipati (2010) argues that two-thirds of Indian colleges and universities do not meet the quality standard.

Furthermore, public institutions cannot provide sufficient higher education to meet demand (Haigh 2010) and the sector has been undergoing rapid privatisation to meet the demand for quality higher education by the Indian middle classes (Agarwal 2009, 66–115). Ownership and financing are two dimensions that distinguish public and private educational institutions (2009, 1–39). Some ‘private’ institutions receive operating funds from the government and are known as ‘private-aided’ institutions, whilst those without aid are fully private institutions. The control of private institutions can be academic as well as administrative, with the latter including financial control when the college is administered by its funding agency (2009, 1–39). Many private institutions are family-owned and for all practical purposes operate as family businesses that meet their expenses through the annual admission/tuition fees paid by students. By law, private institutions are supposedly not-for-profit entities established and functioning through charitable societies or trusts, but very few are genuinely not-for-profit institutions (2009, 66–115 and 39–66). Private institutions often collect exorbitant capitation (fee beyond tuition) and other institutional fees which are not accounted for in their financial accounting. Often this illicit money comes through fraud when the rich obtain admission by flouting admission procedures (2009, 39–66). This anomaly is due to a policy vacuum in the regulatory and funding domains of higher education (2009, 66–115).

Advocates of higher education privatisation claim that it is efficient and ensures quality education that leads to better employment. Whilst efficiency is widely seen as the hallmark of private institutions, it has become diluted in private-aided institutions as a result of complacency (Agarwal 2009, 66–115). Moreover, Agarwal (2009, 402–453) warns that the lack of political will in the past has enabled an expansion of higher education in India in which privatisation is anti-poor and threatens educational equity, because high admission fees limit access to higher education to those with purchasing power. Privatised higher education is in danger of being out of reach for the poor and marginalised (on schools, cf. Jeffery 2005; Drèze and Sen 2013). Agarwal (2009, 402–453) considers that access and equity cannot be ensured unless public policy on education is streamlined. There is, then, a supply-side educational deficit for the poor because they are less able to pursue higher education in private institutions. Further, English-medium education is a kind of class privilege (Faust and Nagar 2001) beyond the reach of the underprivileged. Generally, English-medium schools charge higher fees than vernacular-medium schools, because of the importance of fluency in English for employment. Consequently, English-medium schooling generally reflects a higher economic position.

In Karnataka, the privatisation of higher education started in the early 1950s as an initiative by two dominant caste groups, Vokkaligas and Lingayats, to promote political power and gain material benefits (Kaul 2000). They established several caste colleges and institutions that were managed by caste-based associations. Lately, however, these initiatives have acquired a primarily for-profit character because, in their bid for votes from these caste communities, politicians yield to demands for increased privatisation (Kaul 2000). For instance, according to the AIR (1993 SC 447) judgement, 50% of seats (places) should be reserved by institutions of higher education for meritorious students. Students take a competitive entrance examination to qualify for a so-called ‘free’ seat, and successful candidates are charged lower fees than other students. ‘Meritorious’, of course, itself reflects relative affluence because pupils who could attend good schools and afford extra tuition are more likely to do better in entrance examinations (Kaul 2000). Moreover, due to the state government’s support, more such private caste colleges have been permitted to introduce ‘quota’ seats for political and non-academic reasons (Kaul 2000), thereby further restricting access on the basis of ‘merit’.

## Research methods

This article is based on 26 in-depth interviews conducted with 13 Muslim and 13 Hindu young women pursuing graduate-level education, both in Arts and Science disciplines, at seven colleges: four government-funded institutions (one for women) in west, south-west, east and central Bengaluru; two top-ranking government-aided women’s colleges in west and north-west Bengaluru; and one fully private co-educational college in north Bengaluru. Both government-aided colleges charge high annual admission/tuition fees for Science: rupees ₹24,000 for regular combinations such as Physics, Chemistry and Mathematics, and as much as ₹55,000 for Biotechnology. Arts stream students in private colleges pay less (between ₹12,000 and 20,000) than those in the Science stream, with journalism students paying more than sociology students. In government colleges, the average fee is around ₹2000–3500, irrespective of the stream.

Studying in government-funded colleges is taken as a proxy for the participants’ lower socio-economic status, except for two participants who had enrolled in a government college because of low grades. Private colleges generally have high fees and recruit only students from higher socio-economic backgrounds. Some, however, do cater for different social strata because of their locations and for-profit administration, and recruit students from lower socio-economic backgrounds A more detailed indication of fee structures is elaborated in Table 1, and Table 2 presents the religious, demographic, disciplinary and caste details of the study participants by college type.

**Table 1.** Categorisation of admission fees by college type and stream.

Type	College	College type	Stream	Admission fee (rupees ₹)
Government-aided private/private	College A	Women	Arts	12,000–17,000
	College B	Women	Arts	20,000
			Science	24,000–55,000
	College C	Co-educational	Arts	18,000
Government	College D	Co-educational	Arts	2000–3500
	College E	Women	Arts	1000–,000
	College F	Co-educational	Arts	2000
	College G	Co-educational	Arts	3000

**Table 2.** Distribution of religion, age, college type, discipline and caste.

	Muslim	Hindu
Age		
18	6	5
19	5	3
20+	2	5
Type of college		
Government	6	7
Government-aided private	7	6
Discipline		
Arts	4	6
Science	9	7
Caste		
General		3
OBC		6
SC		4

In-depth interviews based on a detailed interview guideline enabled us to build one-to-one rapport with the participants regarding family and community predispositions towards the pursuit of higher education. In-depth interviews are crucial for capturing people’s individual voices and stories (Hennink, Hutter, and Bailey 2010). Based on the participants’ linguistic comfort, we conducted the interviews in English, Hindi or Kannada. All interviews were conducted by women researchers to enhance the similarity and rapport between the researcher and the participants, and to be congruent with South Asian gender norms. Most of the interviews were conducted by the principal investigator, a 35-year-old middle-class caste Hindu woman, in English, apart from five interviews with Muslim participants whom she interviewed in Hindi. A local female native Kannada-speaker research assistant interviewed five Hindu young women in Kannada. We conducted all the interviews in a quiet space made available on the college premises, such as a library, classroom or laboratory.

At the beginning of the interview, we introduced participants to the research topic, the interview and the use of a recording device, and we assured them of the confidentiality of the information they shared. We asked those who agreed to participate for their written consent. The data were analysed using grounded theory (Glaser and Strauss 1967).

## Young women accessing higher education

### Valuing higher education

Tilak (2007) questions the neglect of secondary and higher education by the government because of assumptions that it is less important than primary education for economic development. Conversely, the Muslim and Hindu study participants alike placed immense value on higher education, which they perceived as an important resource for attaining independence and earning a livelihood. A Hindu OBC participant from a relatively low economic background cited her mother’s example to emphasise how important higher education is for ensuring prized employment. Her father was a manual labourer. Her mother worked in a garment factory: having not studied much, she ended up doing odd jobs and could not provide the participant and her siblings with better life-chances.

Irrespective of economic positioning, Muslim and Hindu participants emphasised the importance of higher education as a safety-net in the event of divorce or widowhood. Drawing from her mother’s experience, a Muslim participant from a middle-class background illustrated how her mother could take care of the family after her father’s death because she was well educated:

For girls, higher education is really important. They can’t depend on husband for everything. See, if husband dies like in my mother’s case she could support family as she is educated and is working.

(Age 20, Muslim, third year of bachelor’s degree, government-aided private college; interview in English^3^)

A Hindu caste participant from a lower socio-economic background also stressed the need for young women to fend for themselves:

We girls get married to some other family. If something happens like divorce or anything bad, we don’t need to be dependent on others. We are educated and are capable of getting a job. Coming back to parents’ house [after divorce] is not always an option because married girls are told about it. Sometimes parents may support. But for the sake of society [the stigma surrounding a woman who lives with her parents after marriage] we have to be independent. With education we can be independent.

(Age 20, Hindu, Okkaliga caste, third year of bachelor’s degree, government college; interview in English)

They did not confine independence to financial independence, but also included developing an identity they can call their own independently of their husband or father, a sentiment that was reiterated by most of the Muslim and Hindu participants. An OBC Hindu participant from an affluent background reasoned that the sense of self-worth comes from the independent identity that young women develop as a result of higher education:

Boys get more opportunity to study than girls. If a girl is educated at least up to degree [graduate], then she can introduce herself to her friends and proudly say ‘I am BA or MA and am working in so-and-so place’. There is a sense of self-respect.

(Age 20, Hindu, Kuruba caste, third year of bachelor’s degree, government-aided private college; interview in English)

Nevertheless, the value placed on modern education tended to be related to the participants’ class position: Muslim and Hindu young women from lower socio-economic backgrounds stressed the importance of higher education for financial independence and a better paying job, whilst participants from wealthier socio-economic backgrounds added that higher education builds self-respect and self-worth. Whilst all the participants stressed the importance of higher education, however, many had confronted several barriers that hampered their ability to realise their educational aspirations to the full.

## Accessing higher education

### Financial constraints

Financial considerations are particularly crucial in determining young women’s educational trajectories, including the duration and quality of their educational experiences. Of the 13 study participants enrolled in four government colleges, five Muslim and five Hindu participants underlined financial insecurity as a barrier to their pursuit of higher education. A Muslim participant explains how financial uncertainty looms large and will probably truncate her aspirations to complete her degree and study further:

Participant: I want to study further because if you do not have higher education then they won’t give us training [prerequisite for National Defense Academy, which she aspires to pursue]. So I have to do my graduation, I want to go beyond, but then I am not sure whether I will be able to.

Interviewer: Why

Participant: My parents won’t allow me. They are like, ‘enough [of education], you have studied enough.’ My father does not have job and my mom is not earning so much, so I need to just stop.

(Age 19, Muslim, first year of bachelor’s degree, government college; interview in English)

Although private colleges are perceived to offer quality education, many choose government colleges because they are cheaper. This is reflected in the experience of a caste Hindu participant who shifted from the private college where she pursued intermediate-level education to a government college for graduation. Her father is a lathe worker and does not have consistent cash income because of frail health, and her mother does not earn enough from her garment factory employment. She explains why she chose to study in a government college:

I studied at college X [for Intermediate], where admission fees was very high, and I didn’t want to burden them [parents] further. The admission fee was around ₹40,000. If I tell my father, how can he make arrangements? It was the last day [for admission], while coming back I saw this college [government] … I went and asked for the admission fee and they told it was ₹2000 and at that time I was happy because ₹2000, I can pay for it, I can study now! Then I told I will study here, my father said OK, but my mother was restricting because it is a Government college [presuming it will compromise the quality of education], then I told her that I liked this college. Then I joined here.

(Age 20, Hindu, Okkaliga caste, second year of bachelor’s degree, government college; interview in English)

She is happy to be able to continue education at the government college amidst her financial troubles, despite her mother’s reservations. Such apprehension about educational quality is not completely unfounded because government colleges are often deficient due to teacher absenteeism and lack of resources. For instance, the Hindu SC participant described inadequacies of the government college where she studies:

We didn’t have teachers for two subjects. We were about to complete a year as January approached, we were scared. This time we don’t have teachers for two subjects, even though tuitions were arranged. With the limited knowledge of Maths, we could not understand anything.

(Age 18, Hindu, Scheduled Caste, first year of bachelor’s degree, government college; interview in Kannada)

This participant struggles to continue studying even at a government college, due to financial hardship. Her father is a manual labourer and her mother works as a domestic help. Her mother borrows money from a moneylender to pay for her college expenses, but the saving grace was a discount of ₹1000 in the admission fee because of her entitlement as an SC to reservations and similar allowances. The parents of a caste Hindu participant could meet the admission fee of ₹2000 only because her mother sold her jewellery. Most of the poorer participants could continue in higher education primarily because they have extremely supportive parents who work hard to fund their daughter’s education and do not hesitate to take loans and sell assets when need arises.

Furthermore, financial hardship can limit subject choice, as for a caste Hindu participant who could not pursue engineering because it is an expensive stream. Similarly, the SC participant could not pursue an integrated and time-effective bachelor’s–master’s programme in a private college because it is too expensive. Financial constraints can also compromise the quality of education, because these economically underprivileged young women cannot afford to seek additional help through tuition. Additionally, those who attend government colleges had attended government schools where they are introduced to English only in the sixth standard. These students face linguistic difficulties when the medium of instruction shifts from vernaculars such as Urdu or Kannada to English in graduate college. This creates an educationally unequal environment where they are more likely to be undervalued when they cannot match up with peers who have studied in private English-medium schools since kindergarten.

Six of the 13 Muslim participants and four of the 13 Hindu participants cited financial hurdles in pursing higher education. Given that our study is based on a small purposively-selected sample, we cannot draw firm conclusions about how Muslim and Hindu young women’s experiences differ either in Bengaluru or in India as a whole. Nevertheless, it is probable that the disproportionate concentration of Muslims among the poor means that Muslim young women are less likely than caste Hindu young women to enjoy equity in terms of pursuing higher education. Be that as it may, our study indicates that financial constraints have limited the choice of college and stream for many of our study participants, Hindu and Muslim alike.

Further, financial insecurity results in living in slums. Whilst this affects young women from poor economic backgrounds in general, this is likely to affect Muslim young women disproportionately. Muslims in India are more urbanised than Hindus and SCs, and urban Muslims are extremely spatially segregated, partly because they face barriers to buying or renting houses in non-Muslim localities (Sachar 2006). The localities where Muslims live generally suffer from greater socio-economic infrastructural disadvantage in terms of educational institutions, medical, postal and telegraph facilities, and so forth, compared with Hindu and SC majority areas (Jeffery 2005; Mohammad-Arif 2012; Sachar 2006; Sahu et al. 2012). With respect to Bengaluru, Mohammed-Arif (2012, 295) has noted the residential clustering of Muslims in different areas of the city, ‘and underprivileged populations tend to live in enclaves such as Tannery road (D.J Halli in particular), Mysore Road, Shivaji Nagar and so on’. Consequently, urban Muslim young women are likely to experience serious supply-side educational constraints (Hasan and Menon 2004, 47–75), as well as living in homes that are congested and lack study space.

A Muslim participant lives with her family in a one-bedroom house in dire poverty because her father is unemployed and her mother sells bangles. Because of lack of space at home, she struggles to study:

In the bedroom there is no window and it’s very dark, we can’t even study inside the room. And kitchen is also very small, obviously you can’t study in the kitchen. There is only one hall where we can study. When I would have just sat down for studying neighbours come. When guests come, it will be distracting. So it’s very difficult to study at home.

(Age 19, Muslim, second year of bachelor’s degree, government college; interview in English)

### Lack of safety for women in public space

There has been a striking increase in the reporting of crime against women in urban India and anxiety about women’s ‘security’ is a crucial hurdle that may hamper young women’s pursuit of higher education. Harassment of young women is likely to occur in any public space and affects those who inhabit affluent spaces as well as slums, whether Hindu or Muslim, whilst they are travelling to educational institutions. In 2013 alone, more than two million Indian women in the age group 15–19 years reported experiencing sexual violence (Raj and McDougal 2014). In India, responsibility for negotiating any ‘danger’ in city life rests on women and they have less access to public space than men (Phadke 2005).

Several Hindu participants from higher socio-economic backgrounds narrated similar challenging experiences about going to college, because of which young women may enforce restrictions on their own movements. Often such restrictions also emanate from parents apprehensive about their daughters’ safety. For instance, a Hindu caste woman studying in a government-aided private college recounted her ordeal of going to college after some rape cases, and the strategies she employed to deal with the threat to women’s safety:

I prefer walking with someone than walking alone due to security reason. We won’t get chased or teased by any guy if we walk together. Ya, I have experienced such loafers just right opposite to our college. Aaa … not physically but ya … they pass comment. Not near my hostel, because I do not step out after 7 o’clock.

(Age 20, Hindu, Brahmin, third year of bachelor’s degree, government college; interview in English)

Participants residing in slums experience greater threats to their safety in public space because such neighbourhoods are dangerous spaces. The Hindu SC young woman living in a slum and studying in a government college illustrates her ordeals on her journey to college due to the regular abuse that she experiences. Yet she decided to continue her education:

Participant: Difficulty is that boys tease while going on the road – ‘Oye [hey] can’t you see, look here. What ya, going alone? Has your friend not come? Did you have lunch?’ While going in the bus, they say ‘bye’. If I don’t say ‘bye’, back they ask, ‘why can’t you say bye’ – this goes on …

Interviewer: How do you feel?

Participant: At that point I feel: I wish I did not have to come to college. Still what to do, difficulties [financial] at home. If I study, then I may get a job and with this hope I come to college.

(Age 18, SC Hindu, first year of bachelor’s degree, government college; interview in Kannada)

Given that Muslims are disproportionately among the poor and likely to be living in slums, the obstacles to travelling to and attending college are generally greater for Muslim than for Hindu young women. A Muslim participant living in a slum was asked by her brother to discontinue her education because there were several *porkey* (anti-social) boys in the locality and their mother did not approve of her continuing in education in such an environment:

Participant: Several *porkey* people live in this area. Therefore my mother is scared for me. She [mother] is interested [in daughter’s education] but she gets scared after looking at the outside world.

Interviewer: Why is she scared?

Participant: All these [rape, harassment] are shown in the serials. She is scared of these *porkey* boys.

(Age 18, Muslim, first year of bachelor’s degree, government college; interview in Hindi)

Since there are hardly any good colleges in or near slums, participants living in slums who wish to study in a better college generally must travel some distance to do so. This acts as another deterrent to achieving their aspirations for higher education. For Muslim young women, the location of colleges in non-Muslim residential areas raises additional fears about attending college. Consequently, Muslim young women may not be able to enhance their potential to its fullest extent.

According to our spatial mapping of our participants’ place of residence and college in which they study, Muslim participants living in an affluent Muslim residential clustering study in an elite government-aided private college located relatively nearby: two Muslim participants from this area travel 3 km to this college, whilst others also from affluent backgrounds travel longer distances (e.g. 9 km, 7 km and 5 km) to study in the same college. The poorer Muslim participants generally study in colleges relatively close to home: one living in an underprivileged Muslim enclave walks to a government college in the same area, another takes a half-hour bus ride to the same college, whilst two others living in another underprivileged Muslim enclave travel shorter distances (e.g. 2.5 km) to the government science college in central Bengaluru. By contrast, Hindu young women tend to travel longer distances: two from wealthy backgrounds each travel about 11 km to study in a government-aided private college, whilst Hindu participants from poorer backgrounds travel even further (e.g. 22 km, 16 km and 12 km) to study in the government science college.

Crucially, Muslim participants studying in colleges close to their residential area are likely to face restrictions on subject choice over and above those due to financial constraints. For instance, although one participant’s parents are keen on educating her, they insisted that she attends a college situated near home where the subject of her choice is not available:

I was interested in doing Computers, but, here we don’t have that option. Only now they introduced Science subjects. I selected subjects: History, Economics, Business Development and Accounts. I took those subjects because my parents asked me. There is no option left before me as my parents will not send me to far-off colleges.

(Age 18, Muslim, first year of bachelor’s degree, government college; interview in English)

Gender bias, gossip and social control 

In light of women’s compromised security in public space, parents often impose gender-biased restrictions on their daughters rather than restrict their sons. In part, this is because parents prioritise the education of sons who are expected to ensure parental old-age security. Investing in girls’ education is often regarded as an expensive liability that does not contribute to their parents’ long-term well-being: for daughters, marriage is their final goal, they leave their natal homes on marriage and need to be provided with a dowry, and there is no point in delaying their marriages beyond the age of 20 or so.

A Muslim participant studying in a government-aided private college is perturbed by her paternal uncles who influence her father against her pursuing higher education:

Participant: My parents are little bit open-minded, but when others [paternal uncle] tell [influence] and when they [parents] listen to others, they start imposing on us. I feel very bad. Always they keep telling my dad ‘why you are sending them for college and all?’ It’s like burden to them, until and unless we get married we are burden on them.

Interviewer: Why burden, what is the reason behind that?

Participant: They have to gather [arrange] dowry and they keep collecting money for that.

(Age 19, Muslim, first year of bachelor’s degree, government-aided private college; interview in English)

Similarly, the SC Hindu participant reports the pressure to marry her to her maternal uncle’s son:

They [relatives] said you have a younger one [participant] give her [in marriage] at least for this proposal. Then my family responded in the negative and said she studies well and has to study further. So they said ‘what you are going to get after educating them? Look at your younger sister [mother’s sister] she has three daughters already two are married. You are the elder sister left with 2 daughters. Look at your younger sister again. She [mother’s sister] is already a grandmother as her children are blessed with kids. Why are you like this!’ My mother replies, that’s their wish [children’s] that’s why I don’t tell my children.

(Age 18, SC Hindu, first year of bachelor’s degree, government college; interview in Kannada)

When financial resources are scarce, families tend to support higher education for sons rather than daughters because they are the future breadwinners. A Muslim participant from a poor economic background complains that her mother does not support her educational aspirations but provides her brother with financial, educational and recreational aids to pursue higher education. She explained that her brother was being treated preferentially because he was valued as a source of old-age security for her parents and investing in his education seemed prudent. Her brother has a designated study corner in their one-bedroom house and everyone is strictly instructed to maintain silence during his study hours. He has a mobile telephone and also a motorbike for commuting to college and tuition. By contrast, the study participant has to study in the kitchen and was often distracted by having to participate in cooking and other household work.

A further consideration is that people in India generally expect women to be less qualified than their husbands. Thus, the educational level of potential husbands impacts on young women’s education when parents try to avoid over-educating them. This is particularly likely to curtail Muslim young women’s higher education. Muslim young men are less likely to attend college than others, largely because of scepticism that their qualifications will translate into formal-sector employment because of discrimination (cf. Basant 2012; Jeffery and Jeffery 1997). For urban Bengaluru, for instance, Mohammed-Arif (2012, 292) notes that ‘there is growing inverse relationship between higher posts and Muslims’.

In addition, conventionally, the patrifocal family was ‘private space’ for women (Agarwal 2000, 283–310) and the family was entrusted with training girls in the altruistic behaviour essential for a future wife and daughter-in-law (Page 2005) and for preserving women’s dignity and chastity (Mukhopadhyay and Seymour 1994). Women’s forays into modern education seem to pose a threat (Chopra 2005) to this established structure, however, because they open a door for women into ‘public space’. A family’s good or bad name is closely linked to how a young woman conducts herself in public, so controls over young women are important to maintain the family’s dignity. Consequently, young women from more affluent backgrounds also experience discrimination within the household.

For instance, a Muslim participant from an affluent background could pursue higher education but was not permitted to study agriculture because her relatives considered it to be a male-dominated field:

Both of us [participant and brother] are given the same importance. But he will take education as career, that one is the difference. He can opt for anything like engineering or anything. That is related to his career. But not in case of me, I have to take job which was not much male-oriented. Like, I wanted to study agriculture, it was my dream. They [parents] told me if you want to do agriculture, then you can do. Some of my relatives have problem with agriculture, because it is male-oriented thing. It has got lots of field-works, it will not suit for girls. It will not be good choice. For that my parents had decided, it’s better not to choose agriculture.

(Age 19, Muslim, second year of bachelor’s degree, government-aided private college; interview in English)

Several other young women described impediments created by relatives and neighbours who try to persuade parents not to permit them to pursue higher education. Two caste Hindu participants from affluent backgrounds, neither a native of Bengaluru (one from a small village and another from a town), are staying in a hostel and as a paying guest respectively. Because of their stay in Bengaluru, both had faced negative remarks about pursuing higher education from their parents’ social circles in their home places. One commented that people feared that her higher education would make her become more assertive:

Participant: They [neighbours and relatives] are not supportive because they feel that I am going to change after attaining education. If a girl ends up learning more, she will know too much and that is not good for her. She will know the truth behind everything in society.

Interviewer: Truth in the sense?

Participant: If girls study more they will speak for themselves. They will get to know what is right and wrong in the society. Those people [who oppose] do not want girls to know more, because with education girls will not keep their mouth shut.

(Age 20, Hindu, Brahmin, second year of bachelor’s degree, government-aided private college; interview in English)

In both cases, either relatives or neighbours had advised the participant’s family to discontinue her higher education because it would make her outspoken or they feared the negative influence of the big city on her. In another instance, the SC Hindu participant described how a neighbour exercised social control by complaining about her conduct to her parents. This had resulted in restrictions on her mobility. By contrast, her brother enjoyed far greater freedom and less scrutiny over his movements, and their parents support his education more than hers, even though his grades were lower than hers:

Participant: They [neighbours] see me on road and immediately complain [that she has been loitering around] to my parents. When I reach home my parents ask me where I went.

Interviewer: What do they say?

Participant: They say that I have gone somewhere and am talking to someone [boy].

This intense social control on her physical mobility has emotionally disturbed her and is hampering her ability to focus on her studies:

Participant: They [parents] don’t leave me alone.

Interviewer: What does it mean?

Participant: If I say that I have to go somewhere, they won’t leave me and even for buying book they won’t allow. They doubt that I will talk with someone [boy].

(Age 18, SC Hindu, first year of bachelor’s degree, government-aided private college; interview in English)

Several Muslim participants also underlined the role of the wider family, neighbours and the community more generally in decisions about their educational trajectory. In a few instances, this raised barriers for the Muslim participants’ pursuit of higher education. One Muslim participant from a low socio-economic background explained how her brother and mother are opposed to her obtaining higher education because studying in college will give her freedom of mobility which is likely to spoil the family’s name. The participant’s father is more supportive towards her obtaining higher education:

They [young women] go and spoil the name outside. He [brother] is scared that even I may become like that. … They have told me that I cannot do graduation and I have to stay at home. My father is suggesting to continue studies through correspondence. But I might not understand accounts through correspondence and language might also be a problem. But my father is supportive of my studies. He says don’t worry. He knows the value of education.

(Age 18, Muslim, first year of bachelor’s degree, government college; interview in English)

Muslims’ residential segregation is a result of financial constraints and discrimination in the housing market, and we have already noted some implications for young Muslim women’s access to higher education and subject choice. Muslim residential clustering also provides a sense of security from close physical proximity (Gayer and Jaffrelot 2012; Jaffrelot 2011; Sachar 2006; Sahu and Hutter 2012, 521–535) in a context of a history of communal violence and (most importantly) the lack of convincing legal action taken against those accused of perpetrating communal violence on them (Jaffrelot 2011). For young Muslim women, however, such security is double-edged. Women are a potent symbol of community dignity and identity. Residential clustering facilitates greater monitoring and control over these young women’s conduct by neighbours and wider kin who can exert pressure on parents to restrict their daughters’ conduct in order to preserve not just the family’s name but also the good reputation of the wider community. Living physically close to fellow Muslims, then, can also impact on Muslim women’s access to higher education and compound the barriers we have already outlined.

Certainly, the Muslim study participants reported experiencing more pressure from beyond the household than the Hindu young women. A Muslim participant from a relatively high socio-economic background describes her father’s strategy to control her conduct in line with the community’s norms of chastity. Although she enjoys more freedom than her relatives, there is a normative expectation to conform because she is being ‘watched’ by others:

Participant: We have so many Muslim families surrounding us in the neighborhood. My family has given me a lot of freedom. The Muslim families living in our area don’t give much freedom to their family members [young women]. The family friends living close to us don’t like a lot of freedom being given to young women. She should be always covered. She can’t come home with friends and lot more. But I do all that. My family has no problem. But sometimes father tell me, that most of the people are watching you. He does not tell not to go, but tells in this manner.

Interviewer: What are those sensitive matters [about which she is likely to be watched]?

Participant: [Smiling] May be not going too close with any of the guys.

Interviewer: Why mingling with boys is not good?

Participant: If girl mingle with them [young men], others think that she has no character, they judge by seeing you. Sometimes they don’t know the reality. But it is difficult to find the groom.

(Age 19, Muslim, second year of bachelor’s degree, private college; interview in English)

Here, the participant’s father stresses her need to conform to codes of conduct such as restricting her interaction with boys and her physical mobility. He is concerned about others in the neighbourhood who might assess his daughter unfavourably and thereby adversely affect her marriage prospects.

The wider family of another Muslim participant belongs to a *Jamaat* ^4^ group and believes in curtailing women’s education and job prospects. None of her cousins was allowed to undertake higher education and a few even had to complete their schooling through correspondence. Her parents have agreed to let her complete her master’s degree. Her mother is supportive about her daughter’s education because she herself did not have the opportunity to continue her education and was married to a relative who is educationally less qualified than she is. The participant, however, could not take up agriculture, her preferred subject, because she would be required to stay away from her family during field visits, which would create avenues for her to enjoy greater freedom. Additionally, the college was co-educational, which was perceived as a threat to her chastity. She expressed her frustration in the following manner:

I am interested in doing agriculture. My dad told me ‘you study whatever you like [supported her choice of subject]. I took the application form, filled it, then I also filled *challan* [money receipt]. I was in the last stage and was about to submit application form when dad’s brother came and asked him ‘why you want to send her [for the course]. For agriculture she has to stay away from house for two to three days in a week. What will you tell others, when your girl is staying away from house?’ My father listened to him blindly and told me ‘don’t go for that’ and ‘just you join this college’ [Biotechnology in a women’s college].

(Age 19, Muslim, first year of bachelor’s degree, private college; interview in English)

If members of the larger family or community uphold restrictive views regarding female education, consulting them before deciding about a young woman’s education can restrict her educational opportunities. This participant could not join a co-educational college. She has, however, been instrumental in convincing her parents to permit her younger sister to do so. The participant’s mother, however, is seeking the opinions of other family members and the participant suspects that this will jeopardise her sister’s chances of enrolling in the college of her choice. She is very critical of how her parents heed the advice of others rather than that of their own daughters:

She [sister] wants to mingle with other people. She doesn’t want to be restricted only to girls’ college. My mom asked for my advice. I told them to send her and told them that even co-ed college is strict and have a dress code. But my mother is asking others. That is the main mistake they have done. They don’t listen to us and pay heed to others’ words. Whatever they tell, they do that and listen to them.

(Age 19, Muslim, first year of bachelor’s degree, government-aided private college; interview in English)

If parents favour education for their daughters yet comply with pressure exerted on them by others due to their greater dependence on their community – as is often crucial for Muslims – their daughters’ pursuit of higher education may be impeded. This might suggest that Muslim families strictly control the conduct of their young women and limit their educational options, but this is not unique to Muslims. Hindu women also experienced this kind of (gendered) social control, although to a lesser degree.

Yet parents are the ultimate authority in deciding a young woman’s future trajectory: either education or marriage. Young women whose parents do not support their education face huge (and possibly insuperable) hurdles in pursuing higher education (as we will discuss in a future article). The participants in this study, however, have all been able to continue their education only due to their fathers’ (and sometimes mothers’) support, at times in the face of the opposition of relatives, neighbours and the larger community.

Despite all of this, the study participants have demonstrated that they are not mere victims of the situation and they have not abandoned their educational aspirations. They have developed creative strategies according to their specific situations, such as the use of secrecy, questioning and negotiation by the less affluent participants, and rationalisation, defiance, compromise and diverting attention by the affluent.

## Conclusion

Popular discourses tend to discount the social and economic disadvantages experienced by Muslims in general, and tend to result in ‘victim-blaming’ discourses. For instance, the root cause of Muslim women’s poor educational attainments is often alleged to be religious conservatism and an Islamic aversion to educating girls. We have not found empirical support for this notion: none of the Muslim participants mentioned any barrier posed by Islam *per se* on their attainment of education, apart from the participant from a conservative *Jamaat* background. Rather, our study indicates that the reasons are much more complex. Irrespective of their religious, social, economic and cultural background, all of our study participants emphasised the importance of higher education for women. Yet several factors constitute significant barriers to attending college, such as the interplay of class and financial position, gender, caste and minority status, residential location and the siting of colleges, women’s ‘security’ in public space, and family and community interventions in decisions about young women’s educational opportunities.

Muslim young women from lower socio-economic backgrounds are likely to face several daunting hurdles in attaining higher education. Financial barriers hamper their chances of entering and continuing higher education. They are likely to reside in poorer neighbourhoods, such as slums, where they face the restrictive influences of neighbours. Because of limited resources, support is often directed towards sons’ education, which compromises daughters’ chances of obtaining higher education. Community-level factors, such as identity politics, Muslims’ spatial segregation and family and community dynamics, are also important. Among these, financial hardship – not religious conservatism – is the most crucial factor in truncating Muslim women’s chances of pursuing higher education, just as is the case for Hindu women from poor backgrounds (a point which is also supported by our research on Hindu and Muslim young women who have discontinued higher education, on which we shall report elsewhere). Young women from affluent backgrounds, whether Hindu or Muslim, are the most likely to pursue higher education, yet fears about their security in public places may delimit their choice of quality education if they are required to study in a college that is for young women only or must opt for subjects taught in a college situated in a ‘safe space’ closer to home. In other words, it is inappropriate to generalise about entire religious communities since the categories ‘Hindu’ or ‘Muslim’ are profoundly fragmented by socio-economic differences. Because Muslims are more concentrated in the ranks of the poor, however, Muslim young women face disproportionately more financial barriers than others and this is crucial for understanding the lower enrolment of Muslim women in higher education.

## Disclosure statement

No potential conflict of interest was reported by the authors.

## Funding

This work was supported by a Wellcome Trust Capacity Strengthening Strategic Award to the Public Health Foundation of India and a consortium of UK universities.

## Notes

1. SCs are recognised by the Constitution of India to be eligible for protective discrimination due to their socio-economic disadvantaged positions. Dalit or ‘oppressed’ is the term that is often used to connote this grouping of castes.

2. We do not have room here to address the question of ‘reservations’ of college places and fee waivers, to which SCs are entitled but Muslims are not. Hasan and Menon (2004) note, however, that Hindu SC young women have made substantial inroads in higher education in the recent past, possibly due to reservation.

3. We have stated the language of interview because English excerpts are the exact words of our interviewees and might not be grammatically correct.

4. A gathering where people listen to Islamic sermons.
